# Is GERD a Factor in Osteonecrosis of the Jaw? Evidence of Pathology Linked to G6PD Deficiency and Sulfomucins

**DOI:** 10.1155/2016/8376979

**Published:** 2016-09-28

**Authors:** Stephanie Seneff, Nancy L. Swanson, Gerald Koenig, Chen Li

**Affiliations:** ^1^Computer Science and Artificial Intelligence Laboratory, MIT, Cambridge, MA 02139, USA; ^2^Abacus Enterprises, Lummi Island, WA, USA; ^3^Health-e-Iron, LLC, 2800 Waymaker Way, No. 12, Austin, TX 78746, USA; ^4^Iron Disorders Institute, Greenville, SC 29615, USA

## Abstract

Osteonecrosis of the jaw (ONJ), a rare side effect of bisphosphonate therapy, is a debilitating disorder with a poorly understood etiology. FDA's Adverse Event Reporting System (FAERS) provides the opportunity to investigate this disease. Our goals were to analyze FAERS data to discover possible relationships between ONJ and specific conditions and drugs and then to consult the scientific literature to deduce biological explanations. Our methodology revealed a very strong association between gastroesophageal reflux and bisphosphonate-induced ONJ, suggesting acidosis as a key factor. Overgrowth of acidophilic species, particularly* Streptococcus mutans*, in the oral microbiome in the context of insufficient acid buffering due to impaired salivary glands maintains the low pH that sustains damage to the mucosa. Significant associations between ONJ and adrenal insufficiency, vitamin C deficiency, and Sjögren's syndrome were found. Glucose 6 phosphate dehydrogenase (G6PD) deficiency can explain much of the pathology. An inability to maintain vitamin C and other antioxidants in the reduced form leads to vascular oxidative damage and impaired adrenal function. Thus, pathogen-induced acidosis, hypoxia, and insufficient antioxidant defenses together induce ONJ. G6PD deficiency and adrenal insufficiency are underlying factors. Impaired supply of adrenal-derived sulfated sterols such as DHEA sulfate may drive the disease process.

## 1. Introduction

Osteonecrosis of the jaw (ONJ) is a debilitating disorder that was originally linked to radiation therapy to treat cancers of the head and neck [1]. However, it later became apparent that ONJ was also a risk factor in cancers not involving radiation of the head/neck region, mainly multiple myeloma and breast cancer. ONJ has now been established as a potential side effect of bisphosphonate (BP) therapy, particularly the nitrogen-containing intravenous BPs (N-IBPs) frequently given to cancer patients. BPs adsorb to bone mineral and interfere with protein prenylation in osteoclasts, reducing bone resorption [2]. Although the evidence is strong in support of a link to ONJ, the mechanism by which this occurs remains unclear, despite much research. In particular, the fact that the jaw is so susceptible to osteonecrosis compared to other bones in the body remains a mystery.

The U.S. Food and Drug administration (FDA) maintains a massive database of adverse drug reactions. In this paper, we investigate the factors linked to ONJ associated with BP therapy by analyzing data from FDA's Adverse Event Reporting System (FAERS). The FAERS database simply lists a set of symptoms and a set of drugs associated with an event with no temporal information. This leaves ambiguous which drugs are causative in the symptom(s) and which are appropriate as treatment of the symptom(s). Nonetheless, FAERS can suggest links among various conditions through statistical averaging of the individual patient data into a collective picture. Comparisons between two groups of patients with or without a specific condition can be powerful for revealing population trends that could be elusive in individual patient data. Our goal here is to quantify interesting statistical correlations and then to use the research literature to establish a plausible biological explanation of our findings. Our discoveries reveal a central pathology involving a disrupted oral microbiome and impaired sulfate supply to body fluids.

The exact pathogenesis of osteonecrosis in general has not yet been elucidated. The main risk factors were thought to be chronic steroid use and alcoholism. Osteonecrosis is usually localized to the hip and jaw. These two bones are both proximal to terminal watershed areas, where blood interfaces with other body fluids (saliva and urine). Large numbers of intravascular thromboses have been found in osteonecrotic femoral heads [3], which links osteonecrosis to a hyperthrombotic state.

Four main theories have been hypothesized to explain the BP-ONJ link. First, BPs accumulate in bone, particularly in the bone-resorbing osteoclasts. Hence, suppression of bone remodeling might induce ONJ [4, 5]. However, there is no evidence of higher accumulation in the jaw compared to other bones [6]. The second hypothesis is that it is caused by an infection in the mouth, particularly* Actinomyces* biofilms, which are often present in ONJ. BPs may increase risk due to immune suppression [7, 8]. A third hypothesis is ischemia following an antiangiogenic effect of BPs, although it does not appear that bisphosphonates necessarily have an antiangiogenic effect on bone tissues [9, 10]. The fourth idea is that local accumulation of BPs leads to mucosal tissue injury and eventually the bone becomes exposed and vulnerable to damage. Although the bone is often exposed with ONJ, this is not an absolute prerequisite for disease [11, 12].

An interesting hypothesis is presented in [13] in which BP-related ONJ may be a consequence of an acidic pH in the mouth, sustained by an overpopulation of the oral cavity microbiome with acidophilic species. If the pH remains low for an extended time period, the microbiota in the mouth transition to acid-tolerant and acid-producing species, including* Actinomyces*, leading to sustained demineralization of the enamel and dentin along with a sustained acidic pH [14]. Acid pH increases the dissociation between BP and hydroxyapatite [15]. A pH as low as 6.2 is not uncommon following an infection, which could be a result of periodontal disease. Low pH activates the nitrogen-containing groups, potentiating a transformation to a toxic metabolite [2, 15, 16]. This could also explain the link to immune suppression, chemotherapy, and diabetes, since all of these would induce disturbances in wound healing, predisposing patients to infection following dental procedures [17–19].

In this paper, we make use of several data subsets of FAERS to tease out various relationships that can help elucidate the underlying pathology of ONJ, as well as explaining the broader pathological context in which ONJ develops. We show that ONJ is associated with vitamin C deficiency (as manifested by symptoms of scurvy), Sjögren's syndrome (a pathology of the salivary glands), and adrenal insufficiency. We also show that ONJ is strongly correlated with acid reflux, another side effect of BP treatment. While such a correlation might be expected simply because BPs cause both side effects [20], we argue here, on the contrary, that gastroesophageal reflux disease (GERD) plays a direct pathological role in ONJ, by inducing an acidic pH in the oral cavity, and this helps explain why BPs cause ONJ.

ONJ has been viewed as an aseptic process. However, a large retrospective analysis of ONJ at the histological/microbiological level revealed that infection plays an important role.* Actinomyces* infection was identified in 69% of 814 patients analyzed in [21].* Streptococcus* was also very commonly found and, to a lesser degree,* Candida*,* Klebsiella*,* Eikenella*, and* Staphylococcus*. This overrepresentation of pathogenic species in the oral microbiome is likely initiated by the acidic environment induced by GERD.

We hypothesize that vitamin C deficiency arises in part because of an impaired ability to maintain vitamin C in the reduced state. Erythrocytes play an essential role in the vasculature in maintaining antioxidant defenses, including NADPH, glutathione (GSH), and ascorbate (vitamin C) [22–24]. They do so by driving glucose through the pentose phosphate pathway (PPP), exploiting glucose as a strong reducing agent. Glucose 6 phosphate dehydrogenase (G6PD) is the rate-limiting enzyme of the PPP. Poorly functioning G6PD can explain not only symptoms of scurvy but also adrenal insufficiency, because vitamin C is present in high concentrations in the adrenal glands, where it enables the synthesis of sulfated hormones such as DHEA sulfate and sulfated corticosteroids. As we will show later, deficiencies in DHEA sulfate are linked to Sjögren's syndrome, and we argue here that this link extends to ONJ pathology.

The PPP is also critical for cellular survival under acidic conditions [25]. Insufficient antioxidant defenses, particularly in the context of acidosis, will lead to widespread damage to cellular proteins, fats, and nucleotides, ultimately explaining necrosis. G6PD deficiency is very common worldwide due to multiple missense mutations [26, 27]. Hence, a plausible explanation for the development of ONJ is impaired G6PD function under conditions of chronic acidosis in the oral cavity. Defects in G6PD could be due to inhibition by environmental toxins [28] as well as to a genetic defect [26, 27] or perhaps both working in concert.

A further component of ONJ is the accumulation of fibrin clots in the jawbone due to impaired plasminogen activity [29–31]. These clots block blood flow in the small capillaries in the bone, leading to a hypoxic condition superimposed on acid stress, along with bone oedema. We will develop this idea more fully in Discussion, where we argue that a state of constant tension between fibrinogen and plasminogen activities drives the observed increased risk to both thrombosis and haemorrhage associated with ONJ. Vitamin C deficiency is an underlying cause of vascular damage due to impaired collagen synthesis.

## 2. Materials and Methods

Each event in the FAERS database lists the observed symptoms along with all drugs administered to the patient. These data are stored using capital letters and we maintain that convention to indicate an exact match from the database. Our methods are based on comparing and contrasting multiple subsets of the FAERS database and recording the frequencies of the appearance of various side effects and administered drugs. We began by finding all cases where osteonecrosis of jaw (ONJ) was mentioned as a side effect between 2002 and 2012. We then identified the top 10 other side effects that were reported along with ONJ. We downloaded all cases where any of these ten side effects occurred in the database over the same time span, whether or not ONJ was also present. We could now divide this subset of FAERS into two distinct sets, those with ONJ (the target dataset) and those without ONJ as a side effect (the comparison dataset). The frequency of the occurrence of a particular drug or symptom in the two datasets could then be compared. If a drug or symptom occurred much more frequently in one dataset over another, an association was suspected and a method of quantifying the discrepancy was formulated. A 95% confidence interval (CI) was determined for each parameter we investigated according to methods described in [32]. Only results that are significant to the 95% CI are reported.

### 2.1. Devising a Score

Following [32], we devised a simple scoring scheme to capture the degree of skew between the two datasets for a given drug or symptom. In particular, we need to know the frequency of occurrence for a given drug or symptom. We therefore define the following:

 
*N*_1_ = the total number of entries in target dataset 1.
 
*N*_2_ = the total number of entries in comparison dataset 2.
 Count 1 = the number of times the drug or side effect was listed in target dataset 1.
 Count 2 = the number of times the drug or side effect was listed in comparison dataset 2.

Then, we have the following:

 
*f*_1_ = Count 1/*N*_1_ = frequency of drug class or side effect in target dataset 1.
 
*f*_2_ = Count 2/*N*_2_ = frequency of drug class or side effect in comparison dataset 2.

If the frequency of the occurrence of a drug or symptom is not associated with either the target dataset or the comparison dataset but is randomly found in both, then the ratio *f*_1_/*f*_2_ will be 1.0. If the drug or symptom is strongly associated with the target dataset, the ratio will be greater than one; if it is more strongly associated with the control dataset, the ratio will be less than one.

We defined a useful metric as

(1) $$\begin{array}{c} score=1000*\left(\;\frac{f_{1}}{\left(f_{1}+f_{2}\right)}\;\right). \end{array}$$

With this schema, a score of 500 indicates that both sets have exactly the same frequency of the factor being measured. A score of 800 means that the target set has four times as many cases with the feature as would be expected based on the distribution in the comparison set. A score of 1000 indicates that all the cases with the given feature were found in the target set (i.e., *f*_2_ = 0).

In order to better generalize our results, we formed a number of categories of drugs or diseases, where we could lump several items into a class because of their shared features. Based on an analysis of the drugs occurring most frequently in our dataset, we organized the main drugs administered into 150 distinct classes, covering a broad spectrum of treatment goals. These goals include anticancer therapy, mood-altering drugs, lipid and blood pressure managing drugs, antacids, vitamins, vaccines, and transfusions. We also formed 49 different categories of diseases which were defined based on simple substring matches. For example, we defined “Lung Disease” as any symptom containing the substring “LUNG” or the substring “PULMONARY.” We defined “Cancer” as a substring match to any of “CANCER,” “TUMOUR,” “TUMOR,” “LYMPHOMA,” or “NEOPLASM,” and we had a separate category for “METASTASIS.”

### 2.2. Detecting Interaction Effects

Though there is strong support that BPs contribute to ONJ, the exact mechanism of action remains elusive [11]. We found a very strong association between BPs and ONJ in the FAERS ONJ dataset. We therefore configured a data subset containing only those patients who were treated with BPs. In comparing this group with the remainder, we noted that acid reflux is strongly associated with BP treatment (score = 860). This is not surprising, as a known side effect of BPs is gastroesophageal irritation [20].

Once we have defined two subsets of interest, we can compare those who have both factors (e.g., BISPHOSPHONATES as a drug and ACID REFLUX as a side effect) with those who have one but not the other, as well as with the general population defined by our top 10 diseases.

More generally, if we find two factors of interest, A and B, we can take our entire dataset of the top 10 diseases associated with ONJ and form three mutually exclusive subsets, namely, A NOT B, B NOT A, and A AND B. We then have three distinct target datasets and one comparison dataset (the entire top 10).

Let *P*_*i*_ = a specific property (e.g., a drug class or a disease class).

Define the following:

 
*f*_0_ = frequency of *P*_*i*_ in the entire dataset.
 
*f*_A_ = frequency of *P*_*i*_ associated with A in the absence of B (A NOT B).
 
*f*_B_ = frequency of *P*_*i*_ associated with B in the absence of A (B NOT A).
 
*f*_AB_ = frequency of *P*_*i*_ associated with the simultaneous presence of A and B.

The frequency ratios *f*_A_/*f*_0_, *f*_B_/*f*_0_, and *f*_AB_/*f*_0_ are unity if there are no interactions and the property *P*_*i*_ is randomly distributed throughout the entire database irrespective of the presence or absence of the properties A and B. In particular, knowing the frequency *f*_A_ of *P*_*i*_ in the target dataset A NOT B and the frequency *f*_B_ of *P*_*i*_ in the target dataset B NOT A, we can predict the expected frequency *f*_AB_ of *P*_*i*_ in the target dataset A AND B.

That is, we have the following:

 Predicted = (*f*_A_/*f*_0_)*∗*(*f*_B_/*f*_0_) = predicted value for frequency of *P*_*i*_ in a target set containing both A and B divided by *f*_0_.

We can compare this value to the observed frequency *f*_AB_: observed = *f*_AB_/*f*_0_ = actual frequency of *P* in target set A AND B divided by *f*_0_. Finally, we define a bias in the ratio of the observed frequency divided by the predicted:

(2) $$\begin{array}{c} bias=\frac{observed}{predicted}=\frac{\left(f_{AB}*f_{0}\right)}{\left(f_{A}*f_{B}\right)}. \end{array}$$

If the bias is 1.0, A and B are independent of each other. The bias is greater than one if A and B are dependent and the presence of one increases the likelihood of the presence of the other. The bias is less than one if A and B are dependent and the presence of one decreases the likelihood of the presence of the other. This parameter captures the anomalous increase or decrease in the frequency of a disease or drug associated with *f*_AB_. For example, if the bias = 4, the property *P*_*i*_ is 4 times as likely to be found in the target set A AND B as would be predicted assuming independence. It turns out to be a very powerful method of extracting synergistic relationships among features in the data, as will be shown in Results.

A simplified example may be helpful to elucidate the concept. Suppose that we have a collection of flowers of ten distinct colors (each flower is a single color). We make several flower arrangements from these. Flowers are placed in each arrangement randomly; however, no arrangement contains two flowers of the same color. The flower arrangements are placed in a field and how often a bee visits each flower arrangement is recorded. The average number of bees per arrangement in a set time period is *f*_0_. We examine separately the following three subgroups: (Y) the flower arrangements that have a yellow flower but do not have a blue flower (*f*_A_), (B) the flower arrangements that have a blue flower but do not have a yellow flower (*f*_B_), and (YB) the flower arrangements that have both a yellow flower and a blue flower (*f*_AB_). Suppose that we find that twice as many bees visit (Y) as observed in the entire population of arrangements and twice as many bees also visit (B). From this we would predict that four times as many bees should visit (YB), but we find that in fact eight times as many visit (YB). From (2), the bias = 2. This means that the two flower colors are synergistically attractive to bees such that even more bees visit the pair of colors than would have been predicted by yellow (Y) and blue (B) subgroups separately.

## 3. Results

In this section, we describe the step-by-step procedures we used to obtain subsets of the FAERS database which could be compared against each other statistically to reveal interesting patterns regarding drugs and side effects linked to ONJ. Furthermore, we identify patterns of side effects consistent with certain complex disorders such as scurvy and Sjögren's syndrome.

All the cases in the FAERS database where ONJ was mentioned as a side effect resulted in a total of 14,054 hits. By tabulating the frequencies of the side effects that were also listed with ONJ, we identified a list of the top 10 side effects linked to ONJ. These were pain, anxiety, osteomyelitis, bone disorder, back pain, osteoarthritis, anaemia, injury, arthralgia, and depression. Half of these are related to bone disorders. We then extracted all the cases where any of these ten side effects were listed, yielding a total of 772,580 entries. Of these, 10,580 included ONJ. We formed two subsets from the group, 10,580 in the ONJ class and 762,002 as the remainder that excluded ONJ (NOT ONJ). We used these two sets to tabulate frequency counts of all the relevant side effects in order to obtain a profile of other features linked to ONJ.

As might be expected, ONJ is strongly associated with diseases of the teeth and gum, as shown in Table 1. The schema adopted in the tables is that a “%” preceding the match-string indicates a substring match, whereas a character string without the “%” requires an exact match. Scores well over 900 were obtained for periodontitis, dental issues, tooth extraction, and any mention of “mucosa.” Rhinitis was also highly overrepresented in the ONJ subset.

Since gingivitis is a common manifestation of vitamin C deficiency, we decided to look more generally at the symptoms linked to scurvy. We mapped the symptoms listed for scurvy in [33] to the nomenclature of the database, with the results shown in Table 2. Nineteen symptoms associated with scurvy were all significantly overrepresented in the ONJ subset compared to NOT ONJ.

Sjögren's syndrome had a score of 920, indicating that it showed up 11.5 times more often in the ONJ dataset. Because Sjögren's is linked to scurvy [34] and apparently also to ONJ, we decided to investigate it further. Sjögren's is an autoimmune disease involving the infiltration of lymphocytes into the salivary and lacrimal glands, resulting in symptoms of dry mouth and dry eye due to insufficient secretion [35]. As shown in Table 3, ONJ exhibits strong associations with Sjögren's itself as well as with nearly all of the symptoms associated with Sjögren's, most impressively with “sialoadenitis” (inflammation of the salivary glands), with a score of 972 (35-fold more common in ONJ compared to NOT ONJ).

In FAERS, each entry has the option to specify which drug is most likely to be the main factor in the reported symptom. The main drug class that we have linked to ONJ is the BPs, particularly the N-IBPs.  Table 4 shows that all but 1% of the ONJ dataset targeted a BP or a similarly-acting drug as the main drug attributed to the observed side effects. Nearly 40% of the cases were linked to the N-IBPs.

Since BPs are used to treat osteoporosis, it is not unexpected that osteoporosis would be highly correlated with ONJ. This is confirmed by the results presented in Table 5 which show that eight out of nine features linked to osteoporosis had scores over 900. This result confirms the robustness of our method in its ability to identify associations in the database.

In addition to Sjögren's syndrome, the symptom profile of ONJ also fits extremely well with the symptoms linked to adrenal insufficiency.  Table 6 shows the skew in the counts for both adrenal insufficiency itself and the symptoms associated with adrenal insufficiency. Out of 13 symptoms, only two had scores below 700: nausea and disorientation. Adrenal insufficiency itself had a score of 944 (17-fold overrepresented in association with ONJ).

The average score for all symptoms that include the substring “ADRENAL” was 965 (28-fold overrepresented in ONJ). Adrenal mass, adrenal adenoma, and adrenal neoplasm all scored above 970.

In looking at the gastrointestinal symptoms linked to ONJ, we were struck by the fact that gastroesophageal reflux disease (GERD), which is very common, was significantly overrepresented in ONJ (919 = 11-fold). This was a very surprising result because GERD has an extremely low association with ONJ by itself. The high frequency turned out to be associated with ONJ only when BPs had been administered.

To further investigate this synergistic effect, we formed three subgroups of our FAERS dataset: (1) BP drug but not GERD, (2) GERD but not BP, and (3) BP AND GERD. We then analyzed these using the mathematical formulation described in Methods. The frequencies of each disease class and each drug class in these three target datasets were analyzed to identify those diseases and drugs, which were remarkably overrepresented when both GERD and BP were present, compared to the predicted value based on frequencies associated with GERD and BP separately.

For symptoms, we set the bias cut-off at a threefold overrepresentation compared to what would be predicted given the frequency in the individual subcategories (BP or GERD). As shown in Table 7, the most outstanding result was that ONJ was extraordinarily overexpressed in association with BP AND GERD compared to the predicted value, by a factor of 196! ONJ is extremely rare in association with GERD unless BPs are administered, in which case GERD increases the risk substantially. Other symptoms that were markedly overrepresented include bone marrow oedema, lymphoedema, and any other forms of oedema, as well as sialoadenitis, anhedonia, and any side effect related to “mucosa.”

In terms of drugs, the anomalously highly overrepresented classes are shown in Table 8. We arbitrarily set the bias cut-off at four times the predicted value, and even with this stringent criterion there were 21 drug classes which were at least fourfold overrepresented in the BP AND GERD set compared to the prediction. Six of these classes were involved with cancer treatment, including various metabolic inhibitors and radiation therapy. Four classes were related to hormones: either hormone treatment or estrogen receptor antagonists. Three classes were antibiotics that are usually reserved for severe hard-to-treat infections. Opium receptor antagonists and smoking cessation agents suggest addiction issues. A very interesting and unexpected result is the enhanced use of Tamiflu to treat flu symptoms (more than 8-fold overrepresented in BP AND GERD). Erythropoietin and transfusion are expected treatments for anemia. The remaining three were immunosuppressants, mouth and throat products, and general anesthetics.

Immunosuppressants may work synergistically with bisphosphonates acting as immune suppressants to induce infection in the mouth associated with ONJ [7, 8], necessitating and explaining the aggressive antibiotic treatments.

While GERD was overrepresented in ONJ by a factor of 11, at the same time inflammatory bowel disease (IBD) was strikingly underrepresented (297). There were zero cases of gingival erythema, gingival abscess, or gingival oedema in the IBD subset, whereas there were over 1000 cases in the comparison set. “TOOTH EXTRACTION,” “PAIN IN JAW,” and “OSTEONECROSIS OF JAW” were all underrepresented in the IBD subset. These observations suggest that IBD and ONJ are to some extent mutually exclusive.

Finally, Table 9 shows that several different types of cancer are strongly associated with ONJ. This is not surprising, as we have already shown not only links to ONJ with the N-IBPs in particular but also the many other classes of cancer treatment drugs shown in Table 8. But this may also imply that cancer is a risk factor for ONJ or that ONJ is a risk factor for cancer. Recurrence of myeloma was 142-fold overrepresented in association with ONJ.

## 4. Discussion

It is well established that BPs may cause ONJ, although the underlying pathology is not understood. BPs were overwhelmingly designated as the most relevant drug in the FAERS dataset in association with ONJ. BPs are widely prescribed to limit bone loss associated with disorders that promote osteoclast-mediated bone resorption, including osteoporosis and metastatic bone cancer.

BPs and N-IBPs work by suppressing the isoprenoid biosynthetic enzyme, farnesyl diphosphate synthase, in the cholesterol biosynthesis pathway [36]. This interferes with geranylgeranylation of the small GTPases, Ras, Rac, Rho, and Cdc42, leading to inhibition of osteoclast activities related to bone turnover. Because they disrupt the cholesterol biosynthesis pathway, they can interfere with the synthesis of sterols [37].

Our statistical analysis of FAERS data reveals a strong signal for scurvy, Sjögren's syndrome, adrenal insufficiency, and GERD. Clearly the adrenal glands are stressed in association with ONJ, and this may well be connected to vitamin C deficiency, since the adrenals have high concentrations of vitamin C which serve an antioxidant effect and catalyze the formation of sulfated sterols [38]. The frequency of severe scurvy is underestimated because diagnoses are often missed, as the symptoms may masquerade as other conditions [39]. Since the adrenal glands are major producers of both sex and stress hormones [40], this also suggests the presence of hormonal insufficiencies in ONJ.

One striking observation we have made is that gastroesophageal reflux disease (GERD) appears not to cause ONJ but to promote the development of ONJ in the context of BP exposure (see Table 7). ONJ is almost never reported in conjunction with GERD except when BPs are also present. More specifically, ONJ was 195-fold more common in association with GERD and BPs than would be expected if there was no interactive component.

Upper GI effects have been well established as a potential side effect of BP therapy [20]. Therefore, the strong correlation between GERD and ONJ may simply reflect the fact that both are side effects of BPs. Nonetheless, we will develop a plausible hypothesis here that GERD is synergistically contributing to the development of ONJ due to increased acidity in the mouth. Acidophilic microbes can maintain the low pH if there is insufficient ionic buffering due to deficient saliva flow. This effect is thus compounded by Sjögren's. Dry mouth is also a side effect of some drugs, and this could cause a similar increase in acidity without explicit Sjögren's. The fact that the mandible is more susceptible to ONJ than the maxilla [41] supports the notion that saliva plays a crucial role in the disease process.

Aminoglycoside antibiotics, glycopeptide antibiotics, and antineoplastic antibiotics were all overrepresented 4-fold to 6-fold in the context of both GERD and BPs compared to the predicted frequency. There are several mechanisms by which antibiotics can lead to acidosis [42]. One is through introducing an imbalance in the gut flora with an overrepresentation of D-lactate-producing* Lactobacillus acidophilus*, along with a decreased representation of lactate-consuming microbes. Disruption of mitochondrial-based aerobic metabolism by certain antimetabolites used to treat both cancer and infection will often favor anaerobic lactate production in both microbes and human cells.

A number of constituents of human saliva are glycosylated [43]. Over sixty different neutral sugars have been identified from the parotid gland, many of which were additionally fucosylated or substituted with sialic acid or sulfate. Salivary sulfomucins are the predominant factor in the defense of the oral cavity against tooth decay [44]. Human salivary sulfomucins are an excellent defense against the common cariogenic bacteria,* S. mutans* and* S. sanguinis*. It has been shown that sulfomucins have 16-fold higher aggregating capacity towards these pathogens than sialomucins. The inhibitory potential against both pathogens is completely destroyed when the sulfomucins are desulfated [44].

Human breast milk contains high concentrations of fucosylated and sialylated oligosaccharides that are theorized to be important for maintaining a healthy balance of gut microbes and fighting off pathogens [45]. One can hypothesize that saliva also similarly provides nutrients to maintain a healthy microbiome in addition to protection from periodontal disease [44]. Reduced salivary flow would compromise this process. Furthermore, it should be noted that swallowed saliva naturally supplies sialomucins and sulfomucins to the abdominal cavity, enhancing defenses against pathogens in the abdominal wall.

Our investigations have led us to propose that a primary factor leading up to ONJ is defective G6PD function in the red blood cells (RBCs). This results in a systemic inability to maintain ascorbate in its antioxidant, reduced form. Since the adrenal glands depend critically on ascorbate to produce sulfated steroid hormones, this pathology results in inadequate supply of DHEA sulfate to the salivary glands. Sjögren's compounds this problem due to its known pathology of deranged sulfotransferase activity in the salivary acinar cells. Defective ascorbate also induces deficiencies in collagen, both in the vasculature and in the oral cavity. In the vasculature, this leads to overexpression of fibrinogen to plug artery leaks and increased risk of fibrin debris infiltrating the capillaries of the jawbone. Fibrin blockage induces hypoxia, which, together with an excessively low pH due in part to acid reflux, causes an environment conducive to the accumulation of a toxic nitrogen-containing metabolites derived from the bisphosphonates in the jawbone. G6PD deficiency in the jaw further aggravates this situation. Meanwhile, ascorbate deficiency in the oral cavity causes degradation in the oral mucosa, and pathogens, particularly those that are attracted to the acidic environment, accelerate mucosal erosion. This exposes the jawbone to the pathogens, enhancing jaw necrosis. On the other hand, erosion of the oral mucosa is beneficial in that it supplies sulfomucins to the gut wall, which may be protective against IBD.

**Figure 1:**
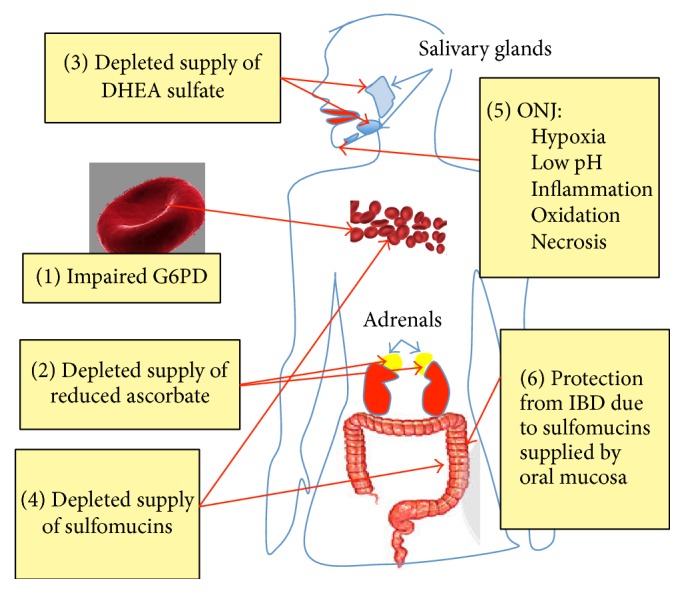
Schematic of the sequential stages of the proposed disease process leading to ONJ and the subsequent protection from IBD due to associated erosion of the oral mucosa.

A summary graphic of our proposed model for the pathological sequelae associated with ONJ is shown in Figure 1. In brief, G6PD deficiency leads to vitamin C deficiency and subsequent adrenal insufficiency. This results in impaired supply of sulfated sterols to the vasculature and the gut. This also leads to Sjögren's syndrome and reduced supply of sulfated sterols to the oral cavity. Concurrent GERD sets up a highly acidic environment in the oral cavity, which, along with hypoxia and other stressors, leads to necrosis of the jaw. Excessive inflammation and oedema in the mouth region result in the breakdown of sulfomucins and sialomucins in the gingiva leading to susceptibility to infection by acid-favoring pathogens but providing some protection from IBD. Support for this theory from the research literature is provided in the following subsections.

### 4.1. Disrupted Oral Microbiome

Infection in the oral cavity is increasingly being recognized as a critical component in ONJ [21]. The population of patients who are exposed to N-BPs is typically immunocompromised, due to malignancy, chemotherapy, or steroid treatments [21]. Individuals treated with N-BP who succumb to ONJ lack immune resiliency, and this impairs their capacity to respond adequately to the immunological stress associated with N-BP treatment. The immune dysfunction could be inherent or acquired through drugs or through illness [46]. Molecular profiling of the oral microbiota in jawbone samples of patients suffering from BP-related ONJ has revealed distinct differences in the colonizing species compared to controls, with a particularly high representation of acid-loving* Streptococcus* [47].

Defects in saliva due to impaired function of the salivary glands is clearly linked to risk of dental caries and is likely also a strong factor in the development of ONJ. Salivary acinar cells normally secrete a fluid that is rich in sodium and chloride, with plasma-like ionic composition [48]. A comparison of the properties of saliva taken from children who were free of caries compared to children with caries revealed several statistically significant differences. The carie-free group had higher mean salivary pH values, higher buffering capacity, lower viscosity, and higher salivary flow rate [49].

Lactoferrin is an iron-binding glycoprotein that can chelate two ferric ions per molecule, and it is present in high concentrations in saliva [50]. By making iron unavailable, it decreases bacterial growth, especially* Streptococcus mutans* and other microbes that are causal in periodontal disease. It also protects from reactive oxygen species due to the Fenton reaction associated with free iron. Lactoferrin's ability to chelate iron is reduced under acidic conditions.

### 4.2. Erythrocytes and Defective G6PD

Erythrocytes (RBCs) play a crucial role in the vasculature of utilizing the reducing power of glucose to maintain NADPH, GSH, and ascorbate (vitamin C) in the reduced form [22–24]. Dehydroascorbate, the oxidized form of ascorbate, does not offer antioxidant protection and may even be prooxidant [51]. RBCs depend upon the PPP and G6PD to utilize glucose as a reducing agent. Lacking mitochondria, RBCs gain reducing power almost exclusively through G6PD. Despite the fact that they are normally protective against oxidation damage, GSH and ascorbate both have a hemolytic effect on G6PD deficient RBCs under conditions of oxidative stress [52].

Thus, in the context of G6PD deficiency, high doses of ascorbic acid can lead to excessive hemolysis [53], and intravenous injection of large doses can be lethal [54]. A mouse study confirmed experimentally that G6PD deficient RBCs incubated with vitamin C experience increased hemolysis [54]. Hemolysis leads to the release of free iron and this increases oxidative stress due to the Fenton reaction.

### 4.3. Glycosaminoglycans and Sulfate

**Figure 2:**
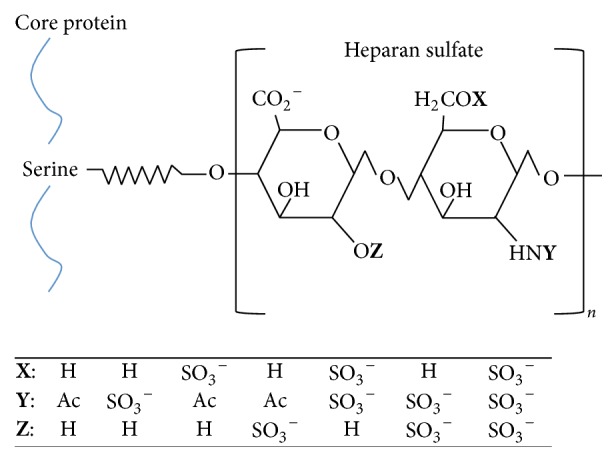
Schematic of complex structure of heparan sulfate chains. [**X**], [**Y**], and [**Z**] can each be just a hydrogen atom or an acetyl group or a sulfate in a complex information-bearing pattern.

Glycosaminoglycans (GAGs) (also called mucopolysaccharides and sulfomucins) are major components of the extracellular matrix (ECM) which undergo significant modifications in content, synthesis, and distribution during acute injury and degenerative diseases. They are long, linear, heterogeneous polysaccharides made up of disaccharide units and modified by acetylation and sulfation at multiple locations in an irregular but not random pattern [55, 56]. Hyaluronic acid (HA) is the only nonsulfated GAG, and heparan sulfate, chondroitin sulfate, dermatan sulfate, and keratin sulfate are the major sulfated GAGs, which are usually attached to a protein core to produce proteoglycans. GAGs occupy the spaces in the extracellular protein matrix of most cells, including the gingival mucosa. Heparan sulfate, in particular, attaches to syndecans in the plasma membrane and binds multiple ligands that play a crucial role in cell-signaling mechanisms. A schematic of the variability in the modifications to heparan sulfate chains is provided in Figure 2.

GAGs are highly dynamic in nature and subject to constant turnover during the life span of the cell. While their varied patterns of sulfation are highly information-bearing, they are not encoded genetically and are therefore difficult to study. However, it is clear that the pattern of sulfation controls their signaling response patterns. For example, the anticoagulant activity of heparan sulfate through antithrombin binding requires a specific sulfation pattern [57].

### 4.4. Collagen, Osteoarthritis, and Wound Healing

Ascorbate plays an essential role in promoting collagen synthesis and maintaining healthy collagen [58]. In the presence of copper as catalyst, ascorbate rapidly induces collagen cross-linking, which strengthens its structure [59]. Various members of the collagen family carry sulfated GAG side chains, including both chondroitin sulfate and heparan sulfate [60]. Nearly half of the molecular weight of collagen XVIII, which is produced by vascular endothelial cells, is a heparan sulfate proteoglycan. Chondroitin sulfate decreases the stiffness of synthetic collagen gels, while unsulfated polysaccharides had no effect [61]. 3-Phosphoadenosine 5-phosphosulfate (PAPS) is the energized form of sulfate which is the rate-limiting factor in sulfation capacity. Mice with an inborn defect in the synthesis of PAPS exhibit impaired bone development due to sulfate deficiency in chondroitin sulfate proteoglycans [62]. The cartilage in joints consists mainly of collagen and the large aggregating proteoglycan, aggrecan. Aggrecan can bind up to 50 times its weight with water, and swelling is normally constrained by the tensile strength of collagen.

Osteoarthritis affects more than 7% of the US population. Osteoarthritis is the result of an imbalance in the turnover of extracellular matrix in cartilage. The proinflammatory agent Interleukin-1 (IL-1) is elevated in osteoarthritic cartilage and is considered a key factor in the pathogenesis [63]. In in vitro studies, IL-1 stimulates the release of matrix sulfated proteoglycans into the culture medium, while inhibiting sulfated proteoglycan synthesis [64]. Osteoarthritis is strongly associated with ONJ in FAERS, with scores of 973 for SPINAL OSTEOARTHRITIS, 968 for NODAL OSTEOARTHRITIS, and 945 for OSTEOARTHRITIS.

According to Rath and Pauling, cardiovascular disease is a direct result of vitamin C deficiency leading to weakening of the collagen-based arterial wall, essentially creating a wound in the vessel [65]. Lipoprotein(a) and fibrin(ogen) accumulate in the artery wall in order to strengthen the weak collagen matrix.

In wound healing, the first step is the infiltration of fibrinogen through extravasation from the disrupted blood vessel to the site of the wound [66]. The coagulation cascade leads to the consolidation of an insoluble fibrin clot to stop the bleeding. This provisional matrix is then infiltrated by fibroblasts which synthesize collagen, catalyzed by ascorbate. Eventually, the fibrin matrix is degraded by plasminogen-induced fibrinolysis and a collagen scar stabilizes and heals the wound. Hypoxia causes increased deposition of ECM components, mainly the collagens. While short-term hypoxia leads to fibrosis due to excess collagen build-up, long-term hypoxia also induces an increase in the synthesis of matrix metalloproteinases (MMPs), leading to a net breakdown of the collagen matrix [67]. This could result in the release of debris that could later clog the capillaries of the jawbone.

### 4.5. Sialic Acid and Sulfate in Mucins

Mucins are high molecular weight glycosylated proteins produced mainly by epithelial tissues and secreted onto mucosal surfaces, such as lining the gingiva and gut epithelial wall. Acidic mucins are categorized as sialomucins or sulfomucins on the basis of whether the terminal unit of the oligosaccharide chain is sialic acid or sulfate [68]. Patients with ulcerative colitis have a lower sulfate content in secreted colonic mucins [69], associated with higher levels of sialic acid.

Rhinitis (strongly overrepresented in association with ONJ) is likely an important mechanism to supply sulfomucins to the gut. Stimulation of mucus synthesis in the rat nasal epithelium by exposure to lipopolysaccharides (LPS) resulted in not only the overproduction of mucus, but also, specifically, the significant overproduction of sulfomucin, 70% of the total compared to only 8.6% neutral glycoprotein [70].

A remarkable recent study has shown that pathogens in the gut are able to exploit sialic acid as a food source, and that this results in excessive growth of selected pathogens following antibiotic exposure [69]. Antibiotic treatment of mice induces a spike in free sialic acid and a consequential overgrowth of* Clostridium difficile* (*C. difficile*). A symbiosis between* Bacteroides thetaiotaomicron* (Bt) and* C. difficile* results in an expansion of* C. difficile* through their ability to consume sialic acid that is enzymatically detached from the mucosal gut lining by Bt. Exogenous dietary administration of free sialic acid can also induce* C. difficile* overgrowth.* CLOSTRIDIUM DIFFICILE* COLITIS was substantially overrepresented in association with ONJ (score of 910). We hypothesize that excessive production of sialic acid in the gingival mucosa followed by its liberation by oral-cavity-resident microbes supplies sialic acid to gut microbes to induce an overgrowth of* C. difficile.*

### 4.6. Sjögren's Syndrome, Salivary Glands, and Sulfate

Sjögren's syndrome is a chronic autoimmune disease in which the white blood cells destroy exocrine glands, specifically the salivary glands and the lacrimal glands [71]. This results in the symptoms dry mouth and dry eyes. It is overwhelmingly a female problem, with a ratio of 9 : 1 female to male [72]. Indeed, 93% of the cases in the ONJ subset with Sjögren's were female. Most women who are affected are in the 40–50-year-old premenopausal age group. It is associated with elevated levels of estrogen in the saliva, along with an elevated ratio of estrogen to androgen.

Primates have a unique capability in the adrenals to release large quantities of the precursor sex steroids DHEA and DHEA sulfate. The salivary glands are able to take up DHEA sulfate and use steroid sulfatases and sulfotransferases as well as steroidogenic enzymes to produce dihydrotestosterone (DHT). Both steroid sulfatase and sulfotransferase are weak and deranged in the salivary glands in association with Sjögren's syndrome [72]. Their location within the cell is abnormal as well as their activity levels. In some cases, sulfotransferase activity was completely absent in the salivary acinar cells.

DHEA sulfate levels are an excellent diagnostic tool for adrenal insufficiency: a normal serum level of DHEA sulfate essentially rules out adrenal insufficiency as a diagnosis [73]. An obvious explanation for the predominance of women with Sjögren's is that men have significant bioavailability of the androgen, testosterone, to help compensate for low DHEA sulfate levels that result from adrenal insufficiency. It is likely that an important part of the role of DHEA sulfate and other sulfated sterols is to deliver sulfate to the receiving cell, as suggested in [74]. The salivary glands can utilize this sulfate by incorporating it into the mucosa.

### 4.7. A Crucial Role for Impaired Fibrinolysis

A key factor in osteonecrosis, whether of the hip or the jaw, is excess platelet activation leading to fibrin clot formation, along with insufficient plasmin(ogen) activity to dissolve the clots.

In 1994, Glueck et al. first proposed a link between hypofibrinolysis and osteonecrosis [29]. In 1997, they proposed that primary, heritable thrombophilia or hypofibrinolysis might cause osteonecrosis of the femur through thrombotic venous occlusion [30]. A follow-on paper in 2007 proposed low molecular weight heparin as a treatment protocol, working as an antithrombotic agent [31]. Heparin is the most highly sulfated organic molecule known to biology. Increased sulfation of the closely related molecules, heparan sulfate and dermatan sulfate, enhances their anticoagulant activities [75, 76]. Recent experiments have shown that heparin causes the formation of thicker fibers in the clot, which makes its structure more open and therefore accessible to fibrinolysis [77]. A later, larger study comparing 45 patients with osteonecrosis to matched controls revealed high incidence of thrombophilic and hypofibrinolytic coagulation abnormalities in the patients [78]. 42% of the patients versus 3% of the controls had high levels of plasminogen activator inhibitor (PAI) activity (*p* < 0.0001).

A well-established risk factor for thrombophilia is elevated serum homocysteine [79]. Glueck et al. report that the likelihood of elevated homocysteine (>13.5 mol/L) was fourfold greater in 36 patients with osteonecrosis compared to controls, and the mean homocysteine level was significantly higher (9.1 versus 7 mol/L) [31]. Homocysteine is a precursor to sulfate, and its oxidation to sulfate depends on access to superoxide, with vitamin C as a catalyst [80]. It is plausible that oxidative damage due to superoxide is a necessary risk under severe sulfate deficiency, whereby homocysteine can be converted locally to sulfate to help correct the deficiency problem. Cancer invasion can be considered as uncontrolled tissue remodelling [81]. Tumor cells release MMPs as well as serine protease plasmin. Efficient inhibition of extracellular proteases, while being attractive as a cancer therapy, has toxic side effects. Mice engineered to have a genetic deficit in the key fibrinolytic protease, plasmin, (PLG−/− mice) developed severe osteoporosis [82]. Although they were indistinguishable from wild-type mice in early life, they eventually developed kyphosis, an unnatural excessive curvature of the spine. KYPHOSIS was overrepresented in the ONJ dataset compared to NOT ONJ by a factor of 55 (see Table 5).

Platelet activation, coagulation, and fibrinolysis play a significant role in metastatic breast cancer [83]. There is a reciprocal interplay between the hemostatic system and the tumor that leads to progression towards metastatic potential. Thrombocytosis is associated with a poor outcome [83, 84]. Indeed, there were 117 cases in our ONJ dataset with THROMBOCYTOSIS, with a score of 958. Thrombin stimulates platelets to release growth factors such as VEGF which promote angiogenesis, providing a blood supply to the tumor [85].

Natural activators of plasmin-mediated fibrinolysis include tissue-type plasminogen activator and urokinase. Tissue-type plasminogen activator binds preferentially to clot-bound plasminogen, whereas urokinase binds equally well to free plasminogen and is thus more likely to induce haemorrhage. Urokinase is overexpressed in association with tumors, and higher levels indicate increased metastatic potential [85]. Both activators, however, also induce platelet activation, leading paradoxically to an increase in fibrin synthesis [86]. We hypothesize that insufficient supply of collagen and sulfomucins to the vasculature is the key underlying pathology leading to defective vascular barrier and overexpression of both fibrinogen and plasminogen, causing constant tension between risks of thrombosis and haemorrhage, both of which are overrepresented in association with ONJ.

Platelet activation can be reduced through prior administration of acetylsialic acid, a component of oligosaccharide chains of mucins, glycoproteins, and glycolipids. This implies that the mucins supplied to the gut via rhinitis and gingival extracellular matrix remodeling may also be protective. Tumors are especially adept at extracellular matrix remodeling, as they chronically both produce and degrade their extracellular matrix structures [87]. We hypothesize that both surgery to remove the tumor and subsequent chemotherapy and radiation therapy cause an increased risk to fibrin clots which will impede blood flow in the microvessels of the jaw, leading to osteonecrosis. This is directly due to the loss of the tumor's supply of sulfated GAGs and plasminogen activator to the vasculature following surgery.

## 5. Conclusion

Our research into FAERS data subsets has led to the observation that ONJ is associated with adrenal insufficiency and symptoms of vitamin C insufficiency. It is also linked to an excess risk to both haemolysis and thrombosis, indicating impaired vascular integrity. Here, we propose that G6PD deficiency may be an important high-level factor in the disease process. We provide evidence that a deficiency in ascorbate due to impaired G6PD function leads to vascular stress due to both impaired collagen cross-linking and depleted sulfate supplies. A deficiency in G6PD initially prevents the circulating erythrocytes from maintaining vitamin C, glutathione, and NADPH in the reduced form, but the deficiency also plays a role in the final pathology in the jaw due to the special requirement for functioning G6PD under acidic conditions.

The adrenal glands depend substantially on reduced vitamin C to produce sulfated sterols, including DHEA sulfate, cortisol sulfate, and the sulfated sex hormones. DHEA sulfate is utilized by the salivary glands in the production of saliva, and Sjögren's syndrome, highly overrepresented in association with ONJ, exhibits a significant pathology in this pathway. Vitamin C is also a catalyst for sulfate synthesis in the artery wall from homocysteine and in the synthesis of sulfated sterols. The maintenance of healthy collagen depends critically on vitamin C, and collagen is also a glycoprotein, bound to sulfated glycosamines that help maintain a tight barrier between the vasculature and the interstitial spaces. When collagen is ineffective as a barrier, fibrin(ogen) can produce a preliminary wound-healing fabric that is later structurally modified and reinforced by newly synthesized collagen anywhere in the vasculature where the barrier is compromised. Heparan sulfate and heparin open up the fibrin matrix to fibrinolysis initiated by plasmin(ogen). Insufficient supplies of heparin due in part to vitamin C deficiency cause an accumulation of circulating fibrous debris in the terminal watershed area of the jaw and kidneys. This effect is compounded by the suppression of fibrinolysis due to chemotherapy and radiation treatment in cancer. This cumulated debris blocks the capillaries, resulting in hypoxia.

In parallel, another contributory factor is the development of GERD as a side effect of BP therapy or for other reasons. Acid reflux combined with low saliva flow (which could be another drug side effect or a result of a disorder such as Sjögren's syndrome) leads to a low pH in the jaw environment, which, combined with hypoxia, greatly stresses the jawbone. The acidic environment in the oral cavity is conducive to the overgrowth of acidophilic pathogens, which support tooth decay and mucosal erosion. The N-IBPs that have lodged in the jaw are metabolized to toxic nitrogen-containing products under acidic conditions, which will enhance the damage due to the inflammatory response to stress.

## Figures and Tables

**Table 1:** Evidence that ONJ is associated with a very strong overrepresentation of diseases of the teeth and gums, as well as rhinitis, compared to NOT ONJ.

Side effect	Count 1	Count 2	*f*_1_/*f*_2_	Score
% periodont	1950	2081	65.7	985
% dental	4281	9029	33.5	971
Tooth extraction	1615	5325	21.7	956
% rhinitis	703	3121	15.9	941
% mucosa	1002	5085	14.2	934
Total count	10580	762002	1.0	500

**Table 2:** Evidence that ONJ is associated with vitamin C deficiency. The symptoms associated with scurvy as defined in [33] are all significantly overrepresented (>95% CI) in association with ONJ compared to NOT ONJ, many by a wide margin.

Side effect	Count 1	Count 2	*f*_1_/*f*_2_	Score
Bleeding gums	841	1792	33.5	971
Impaired wound healing	304	716	30.3	968
Hyperkeratosis	274	925	21.2	955
Joint effusions	401	1742	16.5	943
Inflamed gums	458	2231	14.6	936
Sjögren's	85	531	11.5	920
Ecchymosis	226	1437	11.2	918
Infection	15953	122729	9.3	903
Folliculitis	73	582	9.0	900
Dry eye	331	2796	8.5	895
Dry mouth	608	7289	6.0	857
Petechiae	63	1125	4.0	801
Muscular weakness	734	13614	3.9	795
Anaemia	4602	86759	3.8	792
Haemorrhage	2996	62389	3.4	775
Fatigue	2653	62014	3.1	754
Myalgia	1215	30573	2.9	741
Arthralgia	3012	78226	2.8	734
Depression	3292	106774	2.2	689
Total count	10580	762002	1.0	500

**Table 3:** Evidence that ONJ is linked to Sjögren's syndrome. Both the syndrome itself and the various conditions associated with the syndrome are all overrepresented in association with ONJ compared to NOT ONJ, some by a wide margin of significance.

Side effect	Count 1	Count 2	*f*_1_/*f*_2_	Score
Sialoadenitis	103	209	34.7	972
Vulvovaginal dryness	121	444	19.4	951
% Sjögren's	85	531	11.5	920
Dry eye	331	2796	8.5	895
% lymphoma	196	2289	6.1	860
Dry mouth	608	7289	6.0	857
Dry skin	129	5290	1.8	637
Total count	10580	762002	1.0	500

**Table 4:** Distribution of the main drugs assigned as plausibly causative in the ONJ subset.

Drug class	Count	Percent
Bisphosphonates	6153	58.1%
N-containing IV bisphosphonates	4206	39.8%
Other bone resorption inhibitors	89	0.8%
Other drugs	132	1.2%

Total	10580	100%

**Table 5:** Evidence that ONJ is associated with features strongly linked to osteoporosis. Despite small counts in some cases, all of these features are significantly overrepresented (>95% CI) in association with ONJ compared to NOT ONJ, by a wide margin.

Side effect	Count 1	Count 2	*f*_1_/*f*_2_	Score
Kyphosis	550	702	54.6	982
Postmenopause	30	70	30.3	968
Spinal compression fracture	718	1764	29.3	967
Spinal cord compression	208	587	25.3	962
% fracture	11303	48430	16.5	943
Obesity	427	2080	14.6	936
Type 2 diabetes mellitus	393	2269	12.3	925
Menopause	75	554	9.6	906
Diabetes mellitus	666	7614	6.3	863

**Table 6:** Evidence that ONJ is linked to adrenal insufficiency. Not only is adrenal insufficiency itself highly overrepresented in association with ONJ compared to NOT ONJ, but also the various symptoms associated with adrenal insufficiency are also unusually common among sufferers of ONJ. The score is defined in (1).

Side effect	Count 1	Count 2	*f*_1_/*f*_2_	Score
Any adrenal	591	1541	27.6	965
Addison's disease	35	101	24.6	961
Adrenal insufficiency	133	561	16.9	944
Orthostatic hypotension	216	1592	9.8	907
Myopathy	628	5168	8.7	897
Vertigo	647	6526	7.1	877
Hypoglycaemia	225	2930	5.5	846
Muscular weakness	734	13614	3.9	795
Diarrhea	2265	48901	3.3	769
Fatigue	2653	62014	3.1	754
Vomiting	1594	48934	2.3	701
Nausea	2186	74796	2.1	677
Disorientation	107	4606	1.7	625

**Table 7:** The set of symptom classes that were overrepresented by at least a factor of 3 in the B AND G group compared to the predicted occurrence based on the B NOT G group and the G NOT B group. B: bisphosphonates; G: GERD. As indicated, “MUCOSA” was a substring match, and everything else required exact match. Computation of the score is defined in (1) and computation of the bias is defined in (2).

Side effect	*f*_B_/*f*_0_	*f*_G_/*f*_0_	*f*_BG_/*f*_0_	Bias
Osteonecrosis of jaw	905	15	966	195.85
Bone marrow oedema	858	204	943	10.68
Anhedonia	660	428	851	3.93
% mucosa	738	595	941	3.85
Sialoadenitis	884	395	949	3.74
Lymphoedema	809	546	950	3.73
Oedema	633	675	915	3.00

**Table 8:** List of drug classes that were overrepresented at least fourfold in the B AND G group compared to what is predicted based on the B-only and G-only groups. The ratio was computed according to (2) in the text. B: bisphosphonates; G: GERD.

Side effect	*f*_B_/*f*_0_	*f*_G_/*f*_0_	*f*_BG_/*f*_0_	Bias
Estrogen receptor antagonists	887	50	960	58.09
Mouth and throat products	889	178	971	19.31
General anesthetic	614	442	921	9.25
Mineralocorticoids	697	488	951	8.85
Tamiflu	237	474	695	8.14
Thalidomide derivatives	806	230	900	7.25
Radiation therapy	855	271	937	6.79
Transfusion	539	268	743	6.75
Aminoglycoside antibiotics	554	408	841	6.18
Other immunosuppressants	711	286	851	5.80
Opium receptor antagonists	687	525	928	5.31
Unspecified hormones	861	359	946	5.05
Gonadotropin-releasing hormone	818	200	846	4.89
Proteasome inhibitors	792	298	882	4.62
VEGF/VEGFR inhibitors	612	359	803	4.61
Glycopeptide antibiotics	600	486	866	4.56
Aromatase inhibitors	849	307	915	4.32
Smoking cessation agents	203	329	356	4.43
Antineoplastic antibiotics	729	420	896	4.42
Recombinant human erythropoietin	753	391	889	4.09
Antiviral combinations	170	453	408	4.06

**Table 9:** Overrepresentation in ONJ of cancers typically treated with many of the drugs that were found to be overrepresented in the B AND G group, when compared to NOT ONJ. All of these results are significant at the 95% CI level by a wide margin. B: bisphosphonates; G: GERD.

Side effect	Count 1	Count 2	*f*_1_/*f*_2_	Score
Myeloma recurrence	141	62	141.9	993
Breast cancer recurrent	155	187	57.8	983
Prostate cancer metastatic	144	185	54.6	982
Multiple myeloma	905	1404	44.5	978
Prostate cancer	164	818	14.4	935
Breast cancer metastatic	263	552	33.5	971
Breast cancer	234	2311	7.3	879
